# A local complication-prevention pathway for Impella-supported cardiogenic shock: a single-center experience

**DOI:** 10.1186/s13054-026-06202-7

**Published:** 2026-07-17

**Authors:** Kenji Kanenawa, Akihiro Isotani, Kento Matsui, Keisuke Miyamoto, Katsunori Miyahara, Norihito Oyanagi, Kengo Korai, Toshinari Kaku, Shinichi Tsumaru, Takehiko Matsuo, Norimasa Matsuda, Shinichi Kakumoto, Takenori Domei, Makoto Hyodo, Shinichi Shirai, Kenji Ando, Koh Ono

**Affiliations:** 1 Department of Cardiology, Kokura Memorial Hospital, 3-2-1 Asano Kokurakita-ku, Kitakyushu, Fukuoka, 802-8555 Japan; 2 Department of Cardiovascular Surgery, Kokura Memorial Hospital, Fukuoka, Japan; 3 Department of Anesthesiology and Intensive Care Medicine, Kokura Memorial Hospital, Fukuoka, Japan; 4 Department of Cardiovascular Medicine, Kyoto University Graduate School of Medicine, Kyoto, Japan

Patients with cardiogenic shock requiring temporary mechanical circulatory support are exposed not only to circulatory failure, but also to device-related harm [1]. Although microaxial flow pumps can provide left ventricular unloading and forward-flow support [2], their bedside value may be limited when bleeding, limb ischemia, or hemolysis occurs during support [3]. In our early institutional experience with Impella, several preventable problems clustered around transitions of care: from the catheterization laboratory to the intensive care unit and from device insertion to early stabilization. We therefore reorganized selected local Impella practices as a complication-prevention pathway rather than as device insertion alone.

We describe our experience with 159 patients with cardiogenic shock who received Impella support at Kokura Memorial Hospital between July 2018 and April 2026, after excluding patients treated for indications outside the scope of this local complication-prevention pathway. Because the pathway was introduced stepwise, the experience is described across three periods: before introduction, transition, and full implementation. The pathway was designed to standardize several safety practices across transitions of care. Before leaving the catheterization laboratory, systemic perfusion and lower-limb perfusion were reassessed using lactate values, arterial pressure, pulse examination, skin findings, Doppler signals, and angiography when needed. When limb ischemia was suspected, antegrade limb perfusion was considered early. For Impella CP, access management was standardized with a 16-Fr non-peel-away sheath and continuous side-arm flushing with heparinized saline. In the intensive care unit, lower-limb regional oxygen saturation was monitored continuously in real time during Impella support, with an absolute value < 40% or a relative decrease of approximately 20% prompting bedside reassessment. Hemolysis surveillance was also standardized. Plasma-free hemoglobin was measured once daily, with additional measurements performed when the Impella support level was changed or when hemolysis was clinically suspected. When indicated, Haptoglobin “JB” (Japan Blood Products Organization, Tokyo, Japan) was administered once daily according to prespecified plasma-free hemoglobin thresholds: 2000 units for 20 to < 40 mg/dL and 4000 units for 40 mg/dL or higher. The antithrombotic strategy did not change substantially across the three periods: systemic anticoagulation during Impella support was generally managed with unfractionated heparin targeting an activated clotting time of 160–200 s, and dual antiplatelet therapy was generally used in patients with acute myocardial infarction or those undergoing percutaneous coronary intervention unless contraindicated.

The experience included 73 patients before introduction, 50 during the transition period, and 36 during the full-implementation period. Impella-related Bleeding Academic Research Consortium type 3 bleeding occurred in 26 of 73 patients (35.6%) before introduction, 6 of 50 patients (12.0%) during the transition period, and 4 of 36 patients (11.1%) during the full-implementation period [4]. Detected hemolysis was more frequent during full implementation, consistent with routine plasma-free hemoglobin surveillance. Renal replacement therapy was used in 39 of 73 patients (53.4%), 20 of 50 patients (40.0%), and 12 of 36 patients (33.3%), respectively. Thirty-day mortality also appeared lower across the same periods, occurring in 36 of 73 patients (49.3%), 16 of 50 patients (32.0%), and 8 of 36 patients (22.2%), respectively, but this correspondence is intended to describe a local implementation experience rather than to evaluate treatment efficacy. These results are summarized in Table 1.

These observations should not be interpreted as proof of efficacy for Impella, for the pathway itself, or for any individual component. They are vulnerable to changes over time, case-mix differences, and increasing team experience. Nevertheless, they suggest that the bedside safety of temporary mechanical circulatory support may depend on the surrounding care system as much as on the pump itself [5]. In our experience, the most important change was not a single technical maneuver, but a shared checklist-like culture: reassess perfusion before leaving the catheterization laboratory, search actively for limb ischemia and hemolysis, standardize access care, and make safety checks routine across care transitions. For centers using Impella in cardiogenic shock, a structured complication-prevention pathway may be a practical target for local quality improvement.

**Table 1:** Local Impella-specific complication-prevention pathway and selected complications across three periods

	Before introduction *n* = 73	Transition period *n* = 50	Full-implementation period *n* = 36
**Impella-specific pathway**			
Reassessment perfusion before ICU transfer	Not standardized	Partially introduced	Standardized
Standardized Impella CP access management	Not standardized	Standardized	Standardized
Continuous lower-limb rSO2 monitoring	Not standardized	Partially introduced	Standardized
Routine plasma-free hemoglobin surveillance	Not standardized	Partially introduced	Standardized
Haptoglobin administration protocol based on plasma-free hemoglobin	Not standardized	Partially introduced	Standardized
**Complication**			
Impella-related BARC type 3 bleeding	26 (35.6%)	6 (12.0%)	4 (11.1%)
Detected hemolysis	23 (31.5%)	18 (36.0%)	19 (52.8%)
Renal replacement therapy	39 (53.4%)	20 (40.0%)	12 (33.3%)
Impella-related acute limb ischemia	3 (4.1%)	3 (6.0%)	1 (2.8%)
Stroke	6 (8.2%)	3 (6.0%)	4 (11.4%)

BARC, Bleeding Academic Research Consortium; ICU, intensive care unit; rSO2, regional oxygen saturation. Detected hemolysis reflects events identified through routine surveillance and should not be interpreted as clinically overt hemolysis in all cases

## Data Availability

No datasets were generated or analysed during the current study.
